# Prevalence and Risk Factors of Postpartum Depression in Palestinian Women in the Hebron Governorate, Palestine

**DOI:** 10.2174/0117450179338712240909153229

**Published:** 2024-09-19

**Authors:** Ibtisam Titi, Muna Ahmead, Yehia Abed, Nuha El-Sharif

**Affiliations:** 1 School of Public Health, Al Quds University, Jerusalem, Palestine; 2 Ministry of Health, Hebron, West Bank, Palestine; 3 Juzoor, Gaza, Palestine

**Keywords:** Risk factors, Postpartum depression, Social support, Edinburg Postnatal Depression Scale, Psychosocial factors, Domestic violence

## Abstract

**Background:**

Despite the increased interest from researchers in Postpartum depression (PPD) globally, related studies are limited in Palestine and do not provide a comprehensive understanding of PPD.

**Objective:**

We examined the factors that determine post-partum depression among Palestinian mothers in Hebron governorate.

**Methods:**

A cross-sectional study was conducted in 122 governmental primary healthcare clinics in Hebron Governorate. A convenient sampling method was used to collect data from 435 using a self-administered questionnaire using the Edinburg Postnatal Depression Scale.

**Results:**

The mean EPDS scale score was 10.56 (SD 5.273), and 36.1% had a score of ≥13, indicating moderate to severe depression symptoms. The study results revealed that psychosocial factors were significantly associated with PPDS and play a crucial role in the development of PPD, such as the history of depression, being exposed to domestic violence before and during pregnancy, poor husband support, unplanned pregnancy, fear of infant’s gender, and in addition to anemia than other factors.

**Conclusion:**

A high prevalence of PPD was found among Palestinian women in this study. The study proposes screening women for trauma or domestic violence and assessing their social support, inquiring about pregnancy intention, and discussing family planning. Delivering iron supplements to pregnant or postpartum anemic women is important. Women who have a history of depression, domestic abuse, or lack social support should receive psychological and medical treatment. Mental health services must be included in the after-birth care protocol to train primary health clinic staff to recognize and treat PPD.

## 1 INTRODUCTION

Maternal postpartum depression (PPD) is one of the most common psychiatric complications after childbirth and affects the mother and her child [1-3]. It is defined as a depressive episode that starts within four to six weeks of childbirth and can last for a year [3-5].

The worldwide prevalence of PPD is 17.22% (95% CI: 16.00–18.51). In Western countries, the prevalence of PPD is 14.85%, and it is 19.99% in lower-middle-income countries [6]. Furthermore, PPD varies between Arab countries, with rates of 7.45% in Sudan, 18% in the United Arab Emirates, 37.13% in Bahrain, and 39.78% in Jordan. The discrepancy in the prevalence of PPD can be attributed to cultural disparities, different perspectives on mental health problems and social stigma, socioeconomic status, poverty, inadequate social services, insufficient nutrition, excessive stress, biological variables, healthcare settings, and geographical location [7].

According to the Diagnostic and Statistical Manual of Mental Disorders, fifth edition (DSM-5), PPD develops when a woman meets the criteria for a major depressive episode within the first four weeks after birth [3]. Symptoms of postpartum depression include irritation, sadness, reduced concentration, nausea, and changes in sleeping and eating habits. Furthermore, mothers may experience a lack of energy, persistent exhaustion, and changes in their daily activities as a result of losing interest in caring for their children and families, feeling guilty, and some even considering suicide [8-12].

There are several well-established risk factors associated with the development of postpartum depression (PPD), such as a mother's history of psychological problems, a lack of social support, a troubled relationship with her partner or mother-in-law, a complicated cesarean section, fear of losing the baby or having a female baby, an unplanned pregnancy, and medical or pregnancy complications [6, 13, 14]. Moreover, mothers who encounter stressful life events, low self-esteem, low socioeconomic status, being left alone during birth, having a family history of mental illness, and being exposed to domestic violence are also at risk for PPD [15-20].

Studies have demonstrated that PPD significantly affects the health of parents and their children in general [21, 22]. This includes negative effects on the bonding between mother and infant, as well as lower rates of breastfeeding [23, 24]. In addition, infants of women who do not receive treatment for PPD are more likely to experience chronic problems, such as impaired cognitive functioning, inhibited behavior, emotional maladjustment, aggressive behaviors, externalizing disorders, and psychiatric and medical diseases in adolescence [25, 26].

Successfully managing postpartum depression neces-sitates a comprehensive and multidisciplinary team approach [27]. It was found that both psychological interventions, such as cognitive behavior therapy (CBT) and interpersonal therapy (IPT), and antidepressant medication improve PPD [28]. Health professionals, including obstetricians, psychiatrists, nurses, psycho- logists, and social workers, play a crucial role in the identification, assessment, and support of women experiencing postpartum depression (PPD) [29].

Furthermore, screening tools used in clinical settings are essential for adopting established recommendations and guidelines for treating depression and providing mental health services [30]. In a Malaysian cross-sectional study, it was found that only 25.9% had ever used PPD screening. Also, nurses believed they were responsible for PPD screening, counseling, and referral of mothers for further therapy [31]. These findings highlight the significance of nurses' roles in screening and identifying mothers with PPD.

Women in Palestine face distinct challenges arising from psychosocial, environmental, and political complexities. For instance, the high birth rate of Palestinian women, which stands at 4.468, is influenced by factors, such as early marriage, low divorce rates, and limited use of contraception. Approximately 8.5% of women aged 20-24 in the West Bank were found to be married before the age of 18. Although women have achieved high levels of education, their participation in the workforce remains limited. Within the Palestinian territory under Israeli occupation, nearly half (47%) of girls aged 6-12 suffer from mental or behavioral disorders, which is one of the highest rates in the Eastern Mediterranean Region [32]. Furthermore, women are suffering the effects of domestic violence. Based on the responses of surveyed women in Hebron, gender-based violence is identified as one of the most widespread types of violence in their neighborhoods. The severity of the abuses they observed varied, ranging from verbal to severe bodily harm. These infractions are seldom reported to the authorities [33].

Consequently, the Palestinian healthcare system encounters the difficulty of delivering rapid, adequate, and effective treatment to pregnant women. Also, women-oriented health services mostly focus on family planning and maternal, prenatal, and post-natal care, whereas the provision of psychological and mental intervention is overlooked [32]. Additionally, nurses in Palestine are in charge of after-birth care for mothers and their children. This care includes physical health care for women and their children but does not cover psychological issues like PPD. If symptoms of severe depression are detected, nurses may refer mothers to psychiatrists or psychologists at government primary care mental health clinics. Furthermore, there is no strategy or guideline for PPD screening that nurses may utilize in primary health clinics.

Despite the increased interest from researchers in PPD globally, related studies are limited in Palestine and do not provide a comprehensive understanding of PPD. The prevalence was reported to range from 14% to 34% in governmental primary care centers in Bethlehem and Nablus governorates [34-36]. However, there is a lack of studies on Hebron governorate, which is considered the largest governorate in the West Bank, and its population is 25% of the West Bank population. Therefore, this study aims to examine the prevalence and determinants of post-partum depression among Palestinian mothers residing in Hebron governorate.

## 2 MATERIALS AND METHODS

### 2.1 Study Design and Setting

Observational studies are a key method in research, particularly when performing experiments that are not feasible or unethical. This study utilized a descriptive design, which is a cross-sectional study design. This research design is suitable for performing population-based studies to estimate prevalence and identify risk variables. Moreover, it is advantageous for planning, monitoring, and evaluating public health measures. This study was conducted in 122 governmental primary health care (PHC) clinics in Hebron Governorate, south West Bank. These clinics were allocated into four directorates. The North Hebron directorate is one of four directorates that have 29 PHC clinics. This study covered the five largest clinics among the 29 clinics.

### 2.2 Participants

A convenient sample strategy was utilized to obtain data from all women who visited primary health clinics in the two months between August and September, 2022. Mothers aged 18 years and older who received care at the governmental primary healthcare clinics for postpartum follow-up care after six weeks of birth were invited to participate in the study.

### 2.3 Study Tools

A self-reported questionnaire was employed in the study. The study questionnaire consisted of three sections:

#### 2.3.1 Section One Included a Socio-demographic

Sheet to collect information related to the participants’ socio-demographic variables, *i.e*., mother’s age, years of education, employment status, monthly income, and number of children.

#### 2.3.2 The Second Section of the Questionnaire

The second section of the questionnaire included questions about the mothers’ prenatal history of depression, family history of depression, history of miscarriage, previous history of chronic diseases, current pregnancy history (gravida, complications, gynecology diseases during pregnancy, high-risk pregnancy and anemia during pregnancy), postnatal history (place of delivery, mode of delivery, complications after delivery, infant feeding, infant gender, postnatal anemia), social support (family and partner support), exposure to domestic violence (before pregnancy, during pregnancy, and after delivery), and fear of infant gender.

#### 2.3.3 The Third Section had the Edinburg Postnatal Depression Scale (EPDS)

It is self-administered, and it has been previously translated into Arabic. The EPDS was utilized as a screening tool at 6 weeks after childbirth. This questionnaire was designed by Cox *et al*. [37] for clients to complete to identify signs of depression in women who have just given a child. The screening form consists of 10 items, which can be easily assessed using a 4-point scale, with scores ranging from 0 to 3 for each item. This enables the calculation of summary scores that range from 0 to 30. The scale evaluates the degree of depressive symptoms experienced within the past 7 days.

In previous studies, the EPDS cut-off score ≥11 was shown to have maximum sensitivity and specificity at this value (81% and 88%, respectively) [35, 36, 38] and a cutoff score point of ≥ 13 [39, 40]. The questionnaires were piloted on 45 respondents. The internal consistency, Cronbach’s α, for EPDS in this study was 80%.

### 2.4 Statistical Analysis

Data was entered and analyzed using IBM SPSS Statistics (version 25). Frequencies, means, and standard deviation were used to describe the study data. Bivariate analysis was used to study the relationship between dependent and independent variables. The P-value < 0.05 was considered significant with a 95% confidence interval. Multivariate analysis was performed using logistic regression to eliminate the effect of cofounders. The logistic model includes all variables of interest to be included in the model.

The Helsinki Ethical Committee approved the study. Approval to conduct the study was obtained from the Ministry of Health. Moreover, participants were informed about the aim of the research and signed a consent form.

## 3 RESULTS

### 3.1 Participants

As presented in Table **1**, of the 435 participants, the mean age was 27 years (SD 5.79, range 17 to 45 years), of which 50.1% were in the age group of 23-30 years. Furthermore, 46% of the respondents had received an education ranging from 10 to 12 years, about 80% were unemployed, and 75.4% had a monthly income below $1200. Also, 18.4% had one child, and 23.2% had no children.

### 3.2 Postnatal Depression Symptoms

**Fig. (1):**
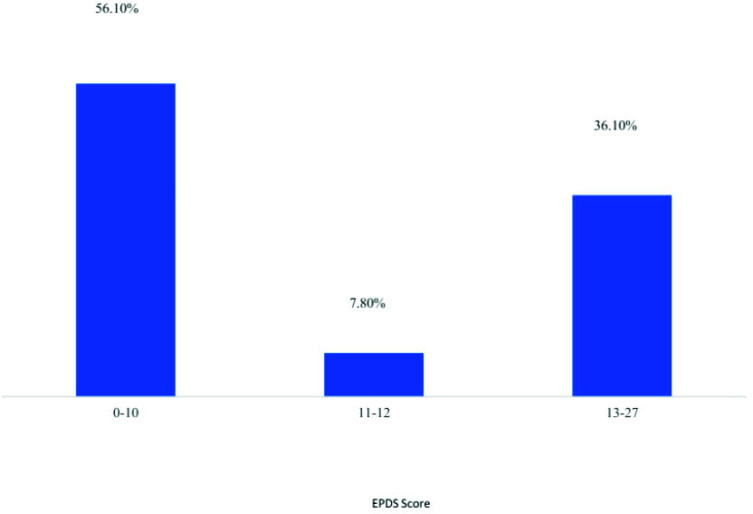
Prevalence of postpartum depression scale EPDS.

The mean EPDS scale score was 10.56 (SD 5.273, 95% CI: 10 -11). The scale ranged from 0 to 27, with a median score of 9.0 and a 75^th^ percentile score of 14.0. Using an EPDS cut-off score of 11, 59.1% scored < 11, while 40.9% scored ≥11. When using a 13-point cut-off, 36.1% had a score of ≥13, indicating moderate to severe depression symptoms (Fig. **1**). Five women reported having thoughts of self-harm “quite often” (question 10), and 14 women reported having these thoughts “sometimes.”

Significant associations were found between EPDS scores and women's age and number of children (p-value < 0.05). Specifically, women aged 23-30 years had the highest EPDS scores, *i.e*., ≥11 (52.4%), as did those having 2-4 children (46.2%). However, there was no statistically significant association between education, employment, monthly income, and EPDS score results (p > 0.05) (Table **2**).

Among women with PPD, 27.7% had a prior history of depression, and 9.4% had a familial history of depression, which was higher compared to those with EPDS scores <11 (p-value < 0.05). Moreover, 25.1% of women with higher EPDS scores had a history of miscarriage compared to 16.4% of women with a lower EPDS score, and the number of pregnancies was also found to be significantly associated with PPD (p-value < 0.05) (Table **3**).

According to Table **4**, 57.9% of women planned their previous pregnancies. However, 37.2% of those with an EPDS score ≥ 11 planned their pregnancy, compared to 74.2% of those with an EPDS score of < 11 (P-value < 0.05). In addition, 14.7% of women with EPDS ≥11 had pregnancy difficulties, 14.7% had gynecological disorders, and 25% were referred to high-risk pregnancy clinics, which was higher than in those without PPD (p-value < 0.05). Furthermore, 28.3% and 38.7% had pre- and postnatal anemia, respectively, which was higher than the comparable group (p-value < 0.05).

As presented in Table **5**, family support and husband support were significantly associated with EPDS scores. Helping in preparing for childbirth, bathing and caring for the baby, and household hose cleaning and care was lower in women with EPDS ≥11 compared to women with lower scores (p-value < 0.05). Additionally, the husband's involvement in making decisions about childbirth, participating in doctor visits, and assisting with infant care was lower in women with EPDS ≥11. Interestingly, experiences of violence during and before pregnancy, along with concerns about the baby's gender, were significantly more common in women with EPDS ≥11.

### 3.3 Multivariate Analysis

Table **6** presents the multivariate logistic regression model comparing women with EPDS scores ≥11 to those with scores lower than 11, revealing several statistically significant factors associated with PPD. A history of emotional problems, exposure to violence before and during pregnancy, and reported poor husband support were highly associated with PPD. Additionally, unplanned pregnancies, concerns about the infant’s gender, and anemia were significantly associated with PPD.

## 4 DISCUSSION

This study aimed to assess the prevalence of postpartum depression (PPD) among women in the Hebron governorate in Palestine. The study's findings revealed that 40.9% of women scored ≥11 on the EPDS, indicating PPD and 36.1% had an EPDS score ≥13, signifying moderate to severe PPD. These figures demonstrate a significant level of PPD, especially when compared to the results of other studies. For example, the occurrence of postpartum depression (PPD) in Western countries usually ranges from 10% to 15%, whereas in lower-middle-income countries, it is approximately 18.6% [6]. In Nablus governorate in Palestine, a study found that 17% of the mothers scored ≥ 10 and were considered depressed, including 8.9% who scored ≥ 13 and were considered to have severe postpartum depression [41]. Furthermore, Alshikh *et al*. [42] found that the overall pooled estimate of the prevalence of postpartum depression was 27% (95% CI 0.19–0.35). Using an EPDS score ≥ 12, the prevalence of PPD was 41% (95% CI 0.14–0.68) and was 25% (95% CI 0.16–0.34) in a subgroup analysis of score ≥ 13. In contrast, a study conducted in UNRWA health centers among Palestine refugee mothers found that women who had given birth at 3-16 weeks had a PPD of 41.9% [43].

Differences in the reporting of the prevalence of postpartum depression (PPD) between the current study and previous studies can be attributed to various factors. First, it was challenging to determine the prevalence of PPD since the DSM-IV's criteria for the time of onset differed from those used in most epidemiological studies [44]. **Second**, women tend to underreport their symptoms of depression [45, 46], as only 20% of female PPD sufferers tell their doctors about their symptoms. **Third**, mothers and medical professionals frequently minimize PPD symptoms as being common, natural side effects of childbirth [44, 47]. **Fourth,** mothers may also be reluctant to discuss their depressive feelings out of fear of stigmatization and the possibility that their depressive symptoms will be interpreted as proof that they are “bad mothers” [44]. **Fifth**, pregnant women face significant challenges in the context of war and military violence. The ongoing political violence and wars in Palestine may affect the prevalence of postpartum depression among Palestinian women due to their exposure to traumatic events. There is a lack of studies that assess the prevalence of PTSD among Palestinian pregnant women. Isosavi *et al*. [48] studied Palestinian women in the Gaza Strip who had prenatal and post-natal traumatic war experiences to identify risk variables for caregiving representations, as well as depression and PTSD-like symptoms. The findings showed that nearly all the mothers reported exposure to war events both before pregnancy and in their postpartum period. Also, 60.0% of the participants reported PPD symptoms in the postnatal period, and approximately 15-16% of the mothers had PTSD symptoms. Muzik *et al*. [49] found that lifetime PTSD symptoms elevated the probability of pregnancy-onset PTSD. Women who experienced a significant rise in PTSD symptoms during pregnancy were more likely to develop postpartum depression and reported the greatest bonding impairment with their newborns at 6 weeks postpartum. Thus, further research is required to assess the relationship between postpartum depression, PTSD, and exposure to war trauma among Palestinian pregnant women.

A high prevalence of PPD poses a serious social problem because of its effects, which include a higher risk of suicide and infanticide [44]. Interestingly, our study results revealed that psychosocial factors were significantly associated with PPDS and play a crucial role in the development of PPD, such as a history of depression, being exposed to domestic violence before and during pregnancy, poor husband support, unplanned pregnancy, fear of the infant’s gender, and in addition to anemia than other factors.

For example, in the current study, a history of depression was a strong risk factor for PPD, similar to the findings of other studies [50-53]. A study by Adamu and Adinew (2018) reported that women who had previously been diagnosed with depression had four times the odds (AOR: 4.2, 95% CI: 2.3-7.8) of reporting postpartum depressive symptoms. Another study in a Palestinian refugee camp found that the main risk factor for PPD was having a history of depression [43]. Unbalanced hormones and stressful times during pregnancy may be contributing factors to depression development [54]. The Palestinian Ministry of Health has developed a postnatal care protocol, but it focuses solely on physical follow-up and ignores mental health issues, such as a history of depression and postpartum depression. In primary care clinics, nurses or midwives are primarily responsible for performing the protocol. It includes visits made following delivery and for up to 42 days afterward, assessing the likelihood of bleeding, symptoms of sepsis or shock, fever, hemoglobin level, vital signs, and babies' health, immunization, and development. Our results might suggest that pregnant women should be screened for depression during the prenatal and postnatal periods and given psychological counseling and treatment in primary health care clinics in Palestine, such as pharmacologic interventions, psychosocial support, professional home visits, and cognitive therapy [47].

Furthermore, in the current study, findings showed that being exposed to domestic violence before and during pregnancy was a strong risk factor for PPD, which is similar to other studies [54, 55]. Domestic violence affected pregnant women emotionally and was strongly linked to postpartum depression in several studies on Ethiopian mothers [54, 56, 57] and Swedish studies [25]. A study by Necho *et al*. [58] revealed that 23.8% of the participants reported experiencing intimate partner violence, with 19% reporting psychological abuse. According to a survey on violence conducted by the Palestine Central Bureau of Statistics, 29.4% of women between the ages of 18 and 64 who were married or had been married had experienced violence in the year before the survey. Among them, 56.6% reported having experienced psychological violence, 17.8% had experienced physical violence, 8.8% had experienced sexual violence, 32.5% had experienced social violence, and 41.1 had experienced economic violence [59]. Women's physical, psychological, mental, and reproductive health are severely affected by violence, suffering from impairments that can lead to death, unwanted pregnancies, depression, and loss of confidence [60]. Therefore, healthcare providers need to screen for a history of trauma or domestic violence and provide appropriate support and resources.

Furthermore, one of the most significant indicators of PPD in the current study is social support. It was found that the husband joining his wife at a doctor's visit, encouraging her to take a break and help with cleaning and cooking, helping her to change her lifestyle, and supporting the woman in caring for the baby decreased the risk of having PPD. Several research studies have demonstrated similar findings [61, 62]. For example, a study in the Nablus governorate in Palestine revealed that husband support was associated with PPD [41]. Similarly, other Arab studies found that not having a husband as a source of support was a significant risk factor for postnatal depression [7, 26, 63]. A study conducted by Yamada *et al*. [64] in Japan indicated that women who received social support from others (*e.g*., mothers and friends) but not from their partners were at high risk for PPD. Thus, it might be advantageous for public health nurses in primary care clinics to ask mothers how much social support they receive from their partners or other people. Nurses can develop a close relationship with mothers who lack social support from their partners or others. Also, spouses may be urged to give their partner adequate support and to take an active role in activities related to childcare [64].

An unplanned pregnancy was another pregnancy-related issue that was found to increase the likelihood of PPD in the current study. This finding is similar to other studies [65, 66]. Necho and colleagues found that women who did not have any plans for pregnancy had a 2.5 times higher chance of developing PPD than those who did [58]. Also, according to a meta-analysis of cohort and case-control studies, unplanned pregnancy was found to be significantly associated with a higher risk of developing PPDS (OR 1.53; 95% CI 1.35-1.74; P < 0.00001) [67]. These findings highlight the necessity of screening for pregnancy intention and integrating family planning and personalized mental health services into primary healthcare to promote maternal mental health. Similarly, fear of a child's gender is another risk factor that significantly correlates with PPD. PPD was linked to having a female baby in Arab culture [16, 68-70] and in other cultures [71]. This result is supported by a study (2020) [58], which found that women who were dissatisfied with the gender of their children had a three times higher chance of developing PPD than women who were pleased with the gender of their children. On the other hand, other studies revealed that the infant's gender had no association with PPD [72-74]. Therefore, an intervention should prioritize increasing social and familial communication as well as decreasing gender preference.

Finally, the findings of this study suggest that early postpartum anemia, which is indicated by low Hb concentration, is a significant risk factor for PPD. The risk for PPD was 6 times higher among women having an antenatal low hemoglobin level. Similar findings were found in a study in the Gaza Strip (2021), which found that body iron status was statistically significantly associated with PPD, and mothers who suffered from iron deficiency (ID) were three times more likely to have PPD (AOR 3.25; P = 0·015) [71]. Fatigue, irritability, and poor concentration have all been linked to anemia [76, 77], and these symptoms may affect how a new mother feels during the postpartum period and how she interacts with her child. Therefore, women who are identified as being anemic during pregnancy or soon after giving birth may be able to prevent PPD with iron supplementation. Moreover, women should receive advice about the dangers of anemia from medical professionals throughout their pregnancies, and they should be reminded to continue eating healthfully even after delivery [78, 79].

### 4.1 Implication for Practice

Palestinian postpartum intervention should focus on mental health issues like postpartum depression (PPD) due to high rates and risk factors. Early detection and treatment are crucial, especially for those with a history of depression, unexpected pregnancies, or gender-related issues. Fathers and mothers should receive postpartum depression and newborn care consultations. A referral mechanism, resources, mental health training, and better communication skills are needed. Public health policy and a nationwide postnatal procedure should prioritize maternal mental health and reproductive decision-making. More study on women's experiences and severity is needed, along with a longitudinal study on PPD's effects on mother and child health. Additionally, for future research on the correlation between postpartum depression (PPD) and violence experienced by women, it is crucial to take into account other types of violence, such as communal violence, workplace violence, and violence and abuse caused by the Israeli occupation.

### 4.2 Study Strengths and Limitations

To the best of our knowledge, this is the first study that investigates a neglected mental health area for women's postnatal care in Hebron governorate, with the highest fertility rate reported in the West Bank. Data was collected from five primary clinics to understand the provision of postnatal mental health interventions.

This study had some limitations. Making causal inferences is hindered by convenience sampling and cross-sectional designs. In addition, the EPDS, a self-reported questionnaire, was used to assess PPD symptoms; however, it cannot be used to make a clinical diagnosis of depression. Furthermore, since only primary health care centers in Hebron governorate were included in the study, it is possible that the results cannot be generalized to other governorates or cities in Palestine.

## CONCLUSION

A high prevalence of postpartum depression (PPD) was found among Palestinian women. A prior history of depression, exposure to violence before and during pregnancy, inadequate spousal support, unintended pregnancy, anxiety over the child's gender, and anemia increase the risk of postpartum depression. The study proposes screening women for trauma or domestic violence and assessing their social support, especially from their husbands, inquiring about pregnancy intention, and discussing family planning. Gender preference should be assessed in routine practice, and delivering iron supplements to pregnant or postpartum anemic women is important. Women who have a history of depression or domestic abuse, lack social support, or have given birth to a girl should receive psychological and medical treatment. These findings also show the significance of healthcare professionals in detecting, monitoring, and treating postpartum depression. Mental health services must be included in the after-birth care protocol to train primary health clinic staff to recognize and treat PPD.

## Figures and Tables

**Table 1:** Selected characteristics of the study population.

**Variable**	**Characteristics**	**Frequency** **(N=435)**	**Percent** **%**
Age (years)	≤ 22	108	24.8
	23-30	218	50.1
	> 30	109	25.1
Years of education	≤ 9	64	14.7
	10-12	200	46.0
	>12	171	39.3
Employment status	Employed	85	19.5
	Unemployed	350	80.5
Income (dollars, $)	≤ 500	70	16.1
	500-1200	258	59.3
	> 1200	107	24.6
Number of children	No children	101	23.2%
	One child	80	18.4%
	2-4	201	46.2%
	≥5	53	12.2%

**Table 2:** Selected sociodemographic characteristics of the study population stratified by EPDS score.

-			**EPDS Score**				-
-	**Total = 435**		**<11** **Total = 244**		**≥11** **Total = 191**		**Chi-square**
**Variable**	**N**	**%**	**N**	**%**	**N**	**%**	**p-value**
**Age years**							
≤ 22	108	24.8%	76	31.1%	32	16.8%	0.001
23-30	218	50.1%	118	48.4%	100	52.4%	
> 30	109	25.1%	50	20.5%	59	30.9%	
**Years of education**							
≤9 years	64	14.7%	40	16.4%	24	12.6%	0.520
10-12	200	46.0%	109	44.7%	91	47.6%	
>12	171	39.3%	95	38.9%	76	39.8%	
**Employment status**							
Employed	85	19.5%	49	20.1%	36	18.8%	0.778
Unemployed	350	80.5%	195	79.9%	155	81.2%	-
**Income dollars, $**							
≤ 500	70	16.1%	37	15.2%	33	17.3%	0.507
500-1200	258	59.3%	142	58.2%	116	60.7%	
> 1200	107	24.6%	65	26.6%	42	22.0%	
**Number of children**							
No children	101	23.2%	69	28.3%	32	16.8%	0.002
One child	80	18.4%	50	20.5%	30	15.7%	
2-4	201	46.2%	104	42.6%	97	50.8%	
≥5	53	12.2%	21	8.6%	32	16.8%	

**Table 3:** Association between prenatal health variables and EPDS score.

-	-		**EPDS Score**				-
	**Total = 435**		**<11** **Total = 244**		**≥11** **Total = 191**		**Chi-square**
	**N**	**%**	**N**	**%**	**N**	**%**	**p-value**
**History of depression**							
Yes	62	14.3%	9	3.70%	53	27.7%	0.000
No	373	85.7%	235	96.3%	138	72.3%	
**Family history of depression**							
Yes	20	4.6%	2	0.80%	18	9.4%	0.000
No	415	95.4%	242	99.2%	173	90.6%	
**History of chronic disease**							
Yes	16	3.7%	9	3.7%	7	3.7%	0.990
No	419	96.3%	235	96.3%	184	96.3%	
Taking medication *							
Yes	55	12.6%	23	9.4%	32	16.8%	0.022
No	380	87.4%	221	90.6%	159	83.2%	
**History of miscarriage**							
Yes	88	20.2%	40	16.4%	48	25.1%	0.024
No	347	79.8%	204	83.6%	143	74.9%	
**Number of pregnancies**							
Primigravida	92	21.1%	65	26.6%	27	14.1%	0.000
1-3 Children	239	54.9%	138	56.6%	101	52.9%	
≥ 4 Children	104	23.9%	41	16.8%	63	33.0%	

Note: *medication means any medication for chronic disease.

**Table 4:** Association between EPDS scale score, pregnancy, and postnatal condition.

-	-		**EPDS score**				-
**Variable**	**Total = 435**		**<11** **Total = 244**		**≥11** **Total = 191**		**Chi-square**
	**N**	**%**	**N**	**%**	**N**	**%**	**P value**
**Planning of present pregnancy**							
Planned	252	57.9%	181	74.2%	71	37.2%	0.000
Unplanned	183	42.1%	63	25.8%	120	62.8%	
**Complication during pregnancy**							
Yes	48	11.0%	20	8.2%	28	14.7%	0.033
No	387	89.0%	224	91.8%	163	85.3%	
**Gynecology diseases during pregnancy**							
Yes	46	10.6%	18	7.4%	28	14.7%	0.014
No	389	89.4%	226	92.6%	163	85.3%	
**Refer to a high-risk pregnancy clinic**							
Yes	72	16.6%	24	9.8%	48	25.1%	0.000
No	363	83.4%	220	90.2%	143	74.9%	
**Type of hospital**							
Governmental	274	63.0%	153	62.7%	121	63.4%	0.890
Private	161	37.0%	91	37.3%	70	36.6%	
**Mode of delivery**							
Vaginal	318	73.1%	179	73.4%	139	72.8%	0.940
Instrumental	21	4.8%	11	4.5%	10	5.2%	
Caesarean section	96	22.1%	54	22.1%	42	22.0%	
**Gestational age at delivery**							
<36	32	7.4%	19	7.8%	13	6.8%	0.843
36-40	381	87.6%	214	87.7%	167	87.4%	
>40	22	5.1%	11	4.5%	11	5.8%	
Having health issues after delivery*							
Yes	19	4.4%	3	1.2%	16	8.4%	0.000
No	416	95.6%	241	98.8%	175	91.6%	
**Gender of baby**	-	-	-	-	-	-	-
Boy	217	49.9%	134	54.9%	83	43.5%	0.060
Girl	212	48.7%	107	43.9%	105	55.0%	
Twins	6	1.4%	3	1.2%	3	1.6%	
**Feeding postnatal**							
Exclusive breastfeeding	194	44.6%	99	40.6%	95	49.7%	0.116
Only bottle-feeding	204	46.9%	125	51.2%	79	41.4%	
Mixed feeding	37	8.5%	20	8.2%	17	8.9%	
**Antenatal hemoglobin value (mg/dl)**							
< 11	86	19.8%	32	13.1%	54	28.3%	0.000
≥ 11	349	80.2%	212	86.9%	137	71.7%	
**Postnatal hemoglobin value**							
< 11	134	30.8%	60	24.6%	74	38.7%	0.002
≥ 11	301	69.2%	184	75.4%	117	61.3%	

**Note:** * Health issues: Infection or sepsis, fever, bleeding, vaginal discharge, weakness, back and muscle pain, severe headache, anemia, and hypertension.

**Table 5:** Family and partner support and life stressors associated with postnatal support.

-			**EPDS Score**					**Chi-square**
-	**Total = 435**		**<11** **Total = 244**		**≥11** **Total = 191**			
**Variables**	N	%	N	%	N	%	**p-value**	
**Family help in preparing for childbirth**								
Yes	315	72.4%	202	82.8%	113	59.2%	0.000	
No	120	27.6%	42	17.2%	78	40.8%		
**Family encouraging antenatal visits**								
Yes	312	71.7%	201	82.4%	111	58.1%	0.000	
No	123	28.3%	43	17.6%	80	41.9%		
**Family help in baby caring: bathing, changing, and feeding**								
Yes	261	60.0%	170	69.7%	91	47.6%	0.000	
No	174	40.0%	74	30.3%	100	52.4%		
**Family help in housework like preparing meals and tidying up**								
Yes	224	51.5%	145	59.4%	79	41.4%	0.000	
No	211	48.5%	99	40.6%	112	58.6%		
**Family financial support**								
Yes	183	42.1%	124	50.8%	59	30.9%	0.000	
No	252	57.9%	120	49.2%	132	69.1%		
**Husband joins you at doctor visits**								
Yes	303	69.7%	201	82.4%	102	53.4%	0.000	
No	132	30.3%	43	17.6%	89	46.6%		
**Husband encourages you to take breaks and help with cleaning and cooking**								
Yes	258	59.3%	176	72.1%	82	42.9%	0.000	
No	177	40.7%	68	27.9%	109	57.1%		
**Husband helps in making decisions about prenatal and postnatal issues**								
Yes	329	75.6%	213	87.3%	116	60.7%	0.000	
No	106	24.4%	31	12.7%	75	39.3%		
**Husband supports in changing lifestyle and quitting smoking**								
Yes	177	40.7%	128	52.5%	49	25.7%	0.000	
No	258	59.3%	116	47.5%	142	74.3%		
**Husbands help feed, change, and bathe your baby**								
Yes	134	30.8%	106	43.4%	28	14.7%	0.000	
No	301	69.2%	138	56.6%	163	85.3%		
**Past experiencing abuse or domestic violence before pregnancy**								
Yes	145	33.3%	30	12.3%	115	60.2%	0.000	
No	290	66.7%	214	87.7%	76	39.8%		
**Exposure to domestic violence during pregnancy**								
Yes	110	25.3%	22	9.0%	88	46.1%	0.000	
No	325	74.7%	222	91.0%	103	53.9%		
**Fear regarding the gender of the child**								
Yes	191	43.9%	84	34.4%	107	56.0%	0.000	
No	244	56.1%	160	65.6%	84	44.0%		

**Table 6:** Multivariate analysis of factors associated with PPDS.

-	Sig.	AOR	95% CI. for AOR	
	-	-	Lower	Upper
Antenatal hemoglobin (<11 mg/dl *vs*. ≥11 mg/dl)	0.000	5.997	2.889	12.448
Women's history of depression (yes *vs*. no)	0.000	9.094	2.930	28.23
Planning of present pregnancy (yes *vs*. no)	0.000	0.223	0.124	0.401
Husband helps in preparing for childbirth (yes *vs*. no)	0.000	0.278	0.141	0.550
Husband joining at doctor’s visits (yes *vs*. no)	0.001	0.328	0.167	0.646
Husband encourages you to take breaks and help with cleaning and cooking (yes *vs*. no)	0.049	0.519	0.270	0.998
Husband supports changing lifestyle and quitting smoking (yes *vs*. no)	0.027	0.476	0.247	0.917
Support in feeding, changing, and bathing your baby (yes *vs*. no)	0.029	0.419	0.192	0.914
Past experiencing abuse or domestic violence before pregnancy (yes *vs*. no)	0.000	5.313	2.719	10.38
Exposure to domestic violence during pregnancy (yes *vs*. no)	0.006	2.836	1.351	5.955
Fear regarding the gender of an infant (yes *vs*. no)	0.000	3.334	1.807	6.152

**Note: **Model control for women’s age and number of children, education level, income level, and employment status. AOR: Adjusted odds ratio,95% CI.: 95% confidence interval.

## Data Availability

The authors confirm that the data supporting the findings of this study are available within the manuscript.

## LIST OF ABBREVIATIONS

PPD: = Postpartum depression
DSM-5: = Diagnostic and Statistical Manual of Mental Disorders, fifth edition
CBT: = Cognitive Behavior Therapy
IPT: = Interpersonal Therapy
PHC: = Primary Health Care

## STANDARDS OF REPORTING

STROBE guidelines were followed.
